# The Current Therapeutic Landscape for Metastatic Prostate Cancer

**DOI:** 10.3390/ph17030351

**Published:** 2024-03-08

**Authors:** Anastasia Bernal, Alivia Jane Bechler, Kabhilan Mohan, Angie Rizzino, Grinu Mathew

**Affiliations:** 1 Eppley Institute for Research in Cancer and Allied Diseases, University of Nebraska Medical Center, Omaha, NE 68106, USA; anbernal@unmc.edu (A.B.); abechler@unmc.edu (A.J.B.); kmohan@unmc.edu (K.M.); arizzino@unmc.edu (A.R.); 2 Fred and Pamela Buffett Cancer Center, University of Nebraska Medical Center, Omaha, NE 68106, USA

**Keywords:** metastatic prostate cancer, castration resistant prostate cancer, neuroendocrine prostate cancer, clinical trials

## Abstract

In 2024, there will be an estimated 1,466,718 cases of prostate cancer (PC) diagnosed globally, of which 299,010 cases are estimated to be from the US. The typical clinical approach for PC involves routine screening, diagnosis, and standard lines of treatment. However, not all patients respond to therapy and are subsequently diagnosed with treatment emergent neuroendocrine prostate cancer (NEPC). There are currently no approved treatments for this form of aggressive PC. In this review, a compilation of the clinical trials regimen to treat late-stage NEPC using novel targets and/or a combination approach is presented. The novel targets assessed include DLL3, EZH2, B7-H3, Aurora-kinase-A (AURKA), receptor tyrosine kinases, PD-L1, and PD-1. Among these, the trials administering drugs Alisertib or Cabozantinib, which target AURKA or receptor tyrosine kinases, respectively, appear to have promising results. The least effective trials appear to be ones that target the immune checkpoint pathways PD-1/PD-L1. Many promising clinical trials are currently in progress. Consequently, the landscape of successful treatment regimens for NEPC is extremely limited. These trial results and the literature on the topic emphasize the need for new preventative measures, diagnostics, disease specific biomarkers, and a thorough clinical understanding of NEPC.

## 1. Introduction

### 1.1. Prostate Cancer Statistics

Prostate cancer (PC) is the second leading cause of cancer-related fatalities among male patients in the United States. According to the American Cancer Society’s projections, 1,466,718 new cases of PC are anticipated worldwide for 2024 [1]. In the United States, approximately 299,010 new cases of PC are anticipated for 2024, and it is predicted that 35,250 of these cases will be fatal [2]. The five-year survival rate for patients with early, localized PC exceeds 99%; however, this rate plummets to 30% for patients with metastasis [3]. For patients with localized low or intermediate risk PC, and on active surveillance (AS; deferred treatment), the fifteen-year treatment free survival rate is approximately 92%. In particular, patients with intermediate risk PC showed significantly lower fifteen-year metastasis-free survival rate, i.e., 10% lower when compared to patients with low risk PC, and a 13% reduction in ten-year overall survival rate when compared to patients with low risk PC [4]. Many of these patients diagnosed with localized PC eventually recur to develop metastatic disease, indicating that there is an unmet need for effective therapies targeting metastatic PC. Additionally, the high incidence of PC and its poor prognosis further emphasize the importance of this health issue, especially for metastatic PC.

### 1.2. Diagnosis of Prostate Cancer

#### 1.2.1. Diagnostic Tests

**Figure 1:**
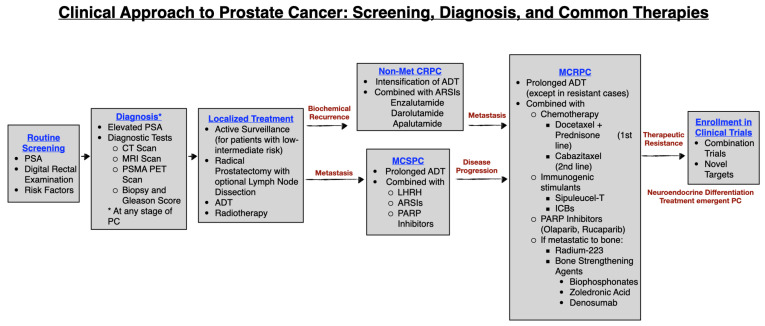
Clinical approach to prostate cancer: screening, diagnosis, and common therapies. A review of the current standard-of-care for PC, discussing diagnosis, standard lines of treatment, therapeutic resistance, and current clinical trials assessing novel therapies for patients with metastatic PC.

Identifying malignant prostate cells requires several orthogonal approaches, especially since patients with early-stage PC display minimal to no symptoms, as summarized in Figure 1. Therefore, there is a pressing need for biomarkers to help stratify PC patients who will progress to more aggressive forms of the disease. A primary and popular screening technique used in PC is the blood-based test for detecting levels of the prostate-specific antigen (PSA). The age at which patients should be screened using PSA is highly debated among clinicians but usually falls between the ages of 40 and 55 [5,6,7]. While PSA serves a valuable role in identifying early signs of PC and monitoring disease progression, the debate surrounding frequent testing or lowering the age requirement has become apparent due to concerns about potential overdiagnosis and unnecessary biopsies [8]. Men with elevated PSA (over 4 ng/mL) require further testing to corroborate the occurrence of PC. In addition, a digital rectal exam (DRE) is often recommended for patients with elevated PSA. Here, the physician examines physical changes of the prostate, such as hardness and irregularity, to predict the incidence of PC [9].

Another important approach is germline testing [10], where cancer-associated genes such as *PTEN*, *TP53*, *RB1*, *BRCA1*, *BRCA2*, *ATM*, *CHEK2*, *MLH1*, *MSH2*, *MSH6*, *PALB2*, *PMS2*, and others are generally screened. These genes are key in the development of several forms of cancer and educate the risk of recurrence, metastasis, and provide insight for personalized treatment plans [11].

#### 1.2.2. Biopsy and Gleason Score

To confirm the incidence and stage of PC, a prostate biopsy sampling the suspicious area is obtained, and examined by a pathologist for abnormalities in cellular morphology and tissue architecture. Healthy cells are uniform and round, while abnormal cells are irregular and span a wide range of shapes [12]. To quantify the progression of disease by degree of cellular differentiation, two independent abnormal areas within the biopsy sample are each given a score between one and five and are added together to yield the Gleason score [12,13,14]. The score is scaled from well differentiated at low numbers (uniform, separate, and rounded glands) to poorly differentiated at high numbers (essentially no glandular differentiation) [14]. A combined score of 6 or lower is low-grade cancer, a score of 7 is intermediate-grade cancer, and a score of 8–10 is high-grade cancer. The predominant tissue grade is listed first in a combined score [14]. Importantly, this distinguishes a Gleason score 7 (4 + 3) tumor to predict a three-fold higher likelihood of aggressive PC compared to a Gleason score 7 (3 + 4) tumor [15]. Thus, Gleason score grading is both an indicator for disease progression and a predictor of aggressive disease.

#### 1.2.3. Histological Classification

During biopsy analysis, a pathologist will also define the type of prostate cell that the cancer arises from. The World Health Organization defines the main categories as epithelial, neuroendocrine, mesenchymal, hematolymphoid, miscellaneous, and metastatic tumors [16]. Each main category holds its own subtypes; epithelial and neuroendocrine are clinically relevant for PC. Among carcinomas arising from the epithelial cells, adenocarcinoma is the most common type of PC. In many cases, adenocarcinoma cells transform into neuroendocrine PC (NEPC), which encompasses various subtypes, each with distinct characteristics and clinical implications. De novo NEPC refers to cases where neuroendocrine features are present from the outset, constituting a small but highly aggressive subset of PC [17]. In contrast, treatment emergent NEPC occurs when standard treatments, such as androgen deprivation therapy (ADT), lead to the development of neuroendocrine features in previously non-neuroendocrine (adenocarcinoma) tumors, representing a challenging scenario in disease management [18].

Within the spectrum of NEPC, small cell neuroendocrine carcinoma (SCNC) and large cell neuroendocrine carcinoma (LCNEC) are the two primary histological subtypes. SCNC are small, round cells defined by characteristic nuclear features and high NE marker expression [17]. SCNC usually arises as treatment emergent NEPC, although a small subset of patients is diagnosed with de novo SCNC. Conversely, LCNEC is an extremely rare de novo NEPC comprising larger cells with prominent nucleoli and shares some molecular features with small cell carcinoma [17]. NEPC, particularly the small cell subtype, is known for its aggressive nature, rapid progression, and limited response to traditional PC treatments, thus emphasizing the importance of classifying morphological and histological features of PC for disease management and improving clinical outcomes. 

#### 1.2.4. Imaging

Imaging tests in conjunction with biopsies are utilized to determine cancer progression and metastasis [19]. A computed tomography (CT) scan can be used to image surrounding lymph nodes and check for metastasis beyond the prostate [20]. To investigate the surrounding bone and prostate adjacent lymph nodes, a magnetic resonance imaging (MRI) scan can be utilized. MRI scans are useful in determining the ideal areas for biopsy, seeing how the cancer has progressed, and if there has been disease recurrence [21]. Another common imaging technique is the positron emission tomography (PET) scan, often used in tandem with MRI or CT scans, revealing metabolic and biochemical function of tissues and organs by tracing the metabolic activity with a radiolabeled compound [22]. This aids in identifying cancerous lesions and in detecting the metastatic sites [22]. Unique to PC, prostate-specific membrane antigen (PSMA) PET scans identify the antigen glutamate carboxypeptidase II, which is expressed abundantly on PC cell membranes. It is useful for assessing metastatic lymph nodes, metastasis in distant organs, and recurrence of cancer, as it can radioactively track the PSMA expressed on PC cells [23].

### 1.3. Common Lines of Treatment: Localized Prostate Cancer

The clinical management of PC is a multifaceted approach, influenced primarily by the stage of disease progression. A comprehensive evaluation consists of a PSA test, followed by histopathological evaluation, and the assessment of genetic and molecular characteristics. Equally significant are patient-specific factors, including family history, life expectancy, personal preferences, and overall health status, all of which collectively inform tailored management of PC [24]. 

Upon initial diagnosis with PC, patients are stratified based on the likelihood of disease stage as low, intermediate, or high-risk, based on the Gleason score and PSA levels. Patients with low and intermediate risk can undergo active surveillance, radiotherapy with or without ADT, or radical prostatectomy. For patients with low risk and low-grade PC, active surveillance becomes an attractive option as it prevents overtreatment in men with slow growing disease. Active surveillance involves scheduled, predefined biannual blood tests to measure PSA levels and an annual DRE. Based on recommendations from the oncologist the patient can opt for MRI-guided biopsy during the follow up; normally performed 1–3 years within the surveillance period [25]. The biopsy samples are then evaluated for different biological parameters such as Gleason score, number of positive biopsies, and percentage of cancer cells within the tumor [26]. 

On the occasion that routine examinations during active surveillance indicate rising PSA or disease progression, patients on active surveillance may opt for surgical resection of the tumor or other therapeutic modalities. Radical prostatectomy is a surgical procedure involving the complete removal of the prostate gland, performed as either open retropubic or perineal, laparoscopic or robotic surgery. Depending on metastatic risk, regional or radical lymphadenectomy may also be considered. For patients with localized PC, radical prostatectomies are often effective and are likely responsible for the >99% five-year survival rate. However, patients with more advanced localized PC are at elevated risk for relapse despite this procedure [24]. Following radical prostatectomy, PSA usually remains undetectable (<0.1 ng/mL). Conversely, a PSA > 0.1 ng/mL serves as an indicative marker for lingering disease, termed biochemical recurrence (BCR). Unfortunately, 20–40% of patients experience BCR within 10 years after a radical proctectomy [27,28]. BCR suggests the need to readjust treatment and often precedes advanced or metastatic progression. 

Generally, patients who underwent radical prostatectomy and maintain undetectable PSA levels do not receive adjuvant radiotherapy. Previously, radiation was considered standard therapy immediately following prostatectomy. However, due to minimal benefits from adjuvant radiotherapy and potential for adverse side effects like urinary incontinence, radiation is now primarily offered once BCR has occurred, termed salvage radiotherapy [29,30]. Salvage radiotherapy has been associated with delayed metastasis and improved survival outcomes in men with detectable PSA levels following radical prostatectomies [31].

Patients with advanced disease are typically recommended for radiotherapy plus ADT or radical prostatectomy with or without lymph node dissection [24]. ADT targets androgen receptor (AR) signaling, a well-known signaling pathway required for PC cell proliferation and survival. ADT significantly lowers testosterone levels, leading to the elimination of PC cells, even in advanced and/or metastatic cases, albeit with diminished effectiveness. In intermediate- to high-risk patients, ADT results in growth repression and improved outcomes when administered as monotherapy and particularly when combined with radiotherapy [32,33]. However, recent evidence suggests controversy on the use of ADT in low-risk patients [33,34]. 

### 1.4. Non-Metastatic Castration Resistant Prostate Cancer (NMCRPC)

When patients continue to have rising PSA levels despite ADT, but no signs of distant metastasis are confirmed with routine imaging (i.e., PSMA-PET scans), they are considered to have non-metastatic CRPC. Treatment at this stage usually involves an early intensification of ADT, often in combination with androgen receptor signaling inhibitors (ARSIs) such as Enzalutamide, Darolutamide, or Apalutamide [35,36,37,38,39]. Notably, one in three patients with non-metastatic CRPC at diagnosis will develop metastasis within 2 years [40]. In spite of this statistic, the metastasis free and overall survival was significantly prolonged in patients with NMCRPC when treated with a next generation androgen receptor inhibitor [36,41,42,43].

### 1.5. Advanced or Metastatic PC

Despite recommendations of routine screening for patients with PC, it is unfortunate that many patients will be diagnosed with metastatic disease, commonly referred to as newly diagnosed metastatic PC (NDMPC). When a patient is initially diagnosed with elevated PSA, and a high Gleason score, the physician assesses if the disease has metastasized beyond the prostate using imaging approaches, such as a PSMA-PET scan. If metastasis has been confirmed, prostatectomy is no longer recommended due to the procedure being invasive and of low benefit for highly advanced metastatic disease. Given the aggressiveness of the disease at this stage, physicians will immediately treat NDMPC with an individualized combination of more intense therapies such as ADT, androgen receptor signaling inhibitors (ARSIs), chemotherapy (Docetaxel), or radiotherapy [29,44,45,46]. 

### 1.6. Metastatic Castration Sensitive Prostate Cancer (MCSPC)

Metastatic castration sensitive PC (MCSPC) is characterized by tumor dissemination from the prostate to distant regions of the body and continued sensitivity to ADT [47]. Considering the cancer is castration sensitive at this stage, most patients will undergo ADT with luteinizing hormone-releasing hormone (LHRH) agonists or antagonists to attack the cancer systematically and use combination therapies if recommended after poor response to initial ADT [48]. When diagnosed at this stage, the predominant treatment consists of medical castration using the LHRH regimen. The treatment with LHRH agonist consists of gradual and prolonged activation of pituitary receptors with LHRH which leads to desensitization and eventual downregulation of gonadotropins and sex steroids such as testosterone. Commonly used LHRH agonists are Leuprolide, Goserelin and Triptorelin. LHRH antagonists are drugs that directly block the pituitary LHRH receptors and cause a rapid reduction in testosterone levels, e.g., drugs such as Degarelix and Relugolix. Other treatment modalities involve combination therapies with ADT such as ARSIs, bone-targeted agents and radionuclides, and Poly ADP Ribose Polymerase (PARP) inhibitors [49]. ARSIs can function by inhibiting androgen synthesis, such as the widely used CYP17 inhibitor Abiraterone acetate. Androgen receptor antagonists (ARAs) are a class of ARSIs that block the ligand binding site of AR thereby inhibiting its subsequent dimerization and nuclear localization. Popular ARAs include second generation ARAs like Enzalutamide and Apalutamide. First generation ARAs include drugs such as bicalutamide and flutamide; however, these do not completely block AR activity. Although used as a popular line of therapy, the use of ARSI is controversial since they are implicated in the transition to castration resistant disease [50]. Other treatment modalities include radionuclides and PARP inhibitors. Radionuclides are effective in targeting metastatic lesions in bone, although they have the risk of reducing healthy bone density. PARP inhibitors target DNA replication in cancer cells, causing reduced proliferation [49]. 

Although many patients respond to standard treatment modalities, some develop therapeutic resistance and eventually develop aggressive, incurable diseases. This may be due a variety of mechanisms such as the epithelial–mesenchymal transition (EMT), the intricacies of the tumor microenvironment, lineage plasticity, epigenetic alterations, genetic mutations, heterogeneous clonality, restoration of AR signaling, and cross-resistance from AR-dependent and AR-independent mechanisms [51,52,53]. 

### 1.7. Metastatic Castration Resistant Prostate Cancer (MCRPC)

After initial responses to systemic treatment, rapid disease progression and/or rising PSA levels may occur despite medical castration [54]. More than 84% of patients are estimated to display metastatic disease at diagnosis of CRPC [40]. Typically, patients that have not already been treated with ADT begin treatment with second generation ARSIs such as Abiraterone or Enzalutamide and may be on this treatment regimen for years [55,56,57]. However, prolonged ADT presents several adverse effects and lower quality of life, increasing risk of cardiovascular diseases and fractures [58,59]. Thus, the continued administration of prolonged ADT must be evaluated carefully. Moreover, some cases of MCRPC develop resistance to ADT by selective survival of AR independent cancer cells such as the neuroendocrine cell type that manifest highly plastic phenotypes [60,61]. 

Concerning castration resistance, it is crucial to have therapeutic avenues that work in tandem with ADT or independently. There are additional approved treatment options for MCRPC, such as chemotherapy, immunogenic stimulants, PARP inhibitors, radium-223 chloride, and bone-targeting agents [49]. Common chemotherapeutic drugs for MCRPC include the taxanes Docetaxel and Cabazitaxel. Docetaxel plus Prednisone is the standard of care for MCRPC, and upon Docetaxel failure, Cabazitaxel is utilized as the second line of treatment [62,63]. Alternative methods include immunogenic stimulants like sipuleucel-T, alongside immune checkpoint blockers (ICBs) such as Pembrolizumab (Keytruda) and Cetrelimab. Immunogenic stimulants aim to engage immune cells to target and attack cancerous cells [64]. Sipuleucel-T, an FDA-approved medication, stands out as a pioneering dendritic cell-based vaccine tailored for treating MCRPC [64]. 

Due to their ability to inhibit cell proliferation, specifically BRCA1- and BRCA2-deficient PC, PARP inhibitors have been shown to aid in the treatment against MCRPC [65]. PARP inhibitors are the first line of clinically approved drugs that take advantage of the phenomenon called synthetic lethality [66]. Synthetic lethality results when functional mutation in a single gene is, by itself, non-lethal; however, these cells are dependent on PARP initiated DNA repair pathway. Therefore, additional inhibition of PARP results in cell death [67,68]. Two PARP inhibitors (Olaparib, Rucaparib) have recently been approved for MCRPC patients with BRCA1 and BRCA2 mutations [65,69]. Thus, genetic testing is utilized to identify whether PARP inhibitors may be an effective option for patients with advanced PC. PARP inhibitors such as Olaparib have shown fruitful results, though necessitating further research [69]. 

Patients with MCRPC also suffer from bone-related damage and are at high risk of spontaneous fractures, which may result in spinal compression. Bone metastasis is a major cause of death, disability, reduced quality of life, and often leads to increased treatment costs for patients with metastatic PC [48,70]. Therefore, bone strengthening agents, such as bisphosphonates (Zoledronic acid) and Denosumab injections were identified and approved by the FDA as an essential part of the treatment regimen for patients with bone metastasis [71,72,73]. Additionally, the alpha emitter radium-223 dichloride (radium-223) has been shown to be effective in targeting bone metastases [74]. A phase II trial, ALSYMPCA, showed an overall survival benefit from treatment with radium-223 regardless of previous Docetaxel treatment and is now recommended for MCRPC patients with bone metastasis [74]. Lutetium-177-PSMA-617 (PLUVICTO^(R)^) is another clinically approved therapy that uses an antibody against PSMA [75,76]. This radioligand therapy delivers beta-particle radiation to cancer cells expressing PSMA, ensuring high selectivity to the cancer lesions. PSMA-PET imaging can be used to select for PSMA positivity and thus acts as a second modality in Lu-177 PSMA therapy.

### 1.8. Metastatic NEPC (MNEPC)

Among the mechanisms of tumor survival, neuroendocrine (NE) differentiation has emerged as a significant contributor towards treatment resistant disease progression. Increasing evidence supports that lineage reprogramming from adenocarcinoma, which is AR dependent, to the neuroendocrine cell type that typically lacks AR signaling, acts as a mechanism of resistance manifested by NEPC. Lineage reprogramming occurs when the differentiation of a mature somatic cell into another somatic cell happens without reverting to an earlier stem cell fate [18]. Termed as “treatment emergent NEPC”, small cell neuroendocrine carcinoma of the prostate often arises in response to selective pressure under hormonal therapy like ADT. Thus, resistance to standard therapies contributes to the molecular landscape of NEPC and presents the need to adjust treatment regimens unique to treating or preventing aggressive MNEPC. 

## 2. Clinical Trials for Late-Stage Neuroendocrine Prostate Cancer

Clinical trials play a crucial role in advancing our understanding of effective treatments for diseases like PC. The trials assessed in this review are referenced in terms of development in the clinical approval process, phase I trials indicating the early stages and phase III indicating the later stages. Phase I primarily focuses on assessing the safety of the new intervention and typically involves only a few individuals. Once phase I trials have shown some promise or benefit, phase II trials expand the study to a slightly larger group for a more comprehensive evaluation of the effectiveness and safety of the treatment regimen. Phase III involves a few hundred participants to monitor and compare the new treatment to existing standard treatments, and the clinically beneficial parameters are only held in comparison to the control arm. Finally, phase IV has an even larger number of participants, typically in the thousands, and assesses the long-term safety and side effects of the new treatment. 

The trials are categorized based on combinations of established standard lines of therapy, combination therapy, or novel agents, each associated with their respective phases. Where results are available, treatment efficacy is assessed through clinically beneficial parameters such as overall response rate (ORR), progression-free survival (PFS), and overall survival (OS). ORR gives the percentage of patients with partial or complete therapeutic response. PFS gives the average time, in months, that patients showed no disease progression, typically monitored by PSA levels and/or imaging. OS measures the average length of time that patients survive after starting the trial.

### 2.1. Combination Clinical Trials

**Figure 2:**
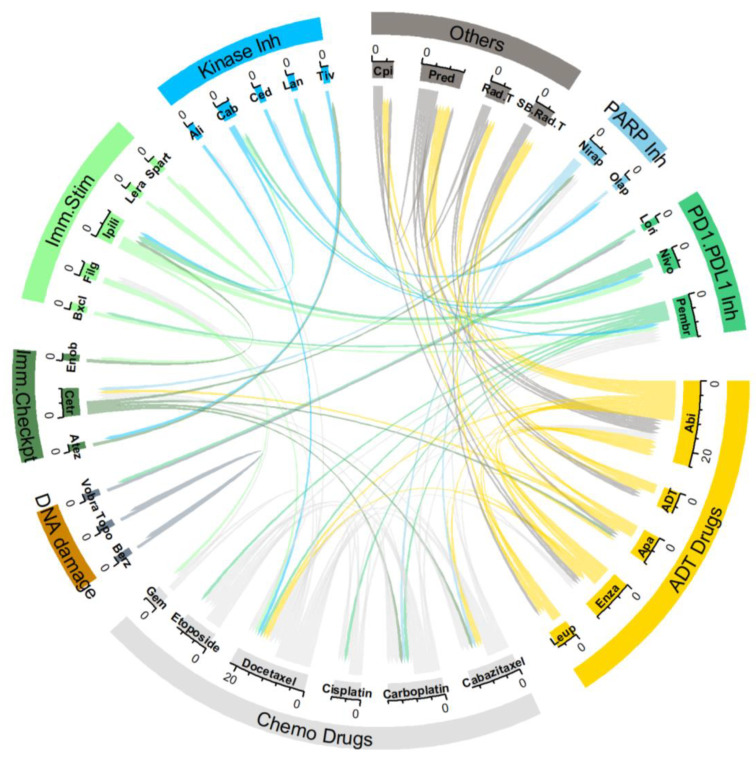
Circos plot of drug combinations in clinical trials for NEPC. The drugs are classified into broad groups based on their expected functions and linked based on combination use in a clinical trial. Each drug pair has both an outgoing and incoming link. Outgoing links are depicted with a flat end while incoming links are depicted with arrows that terminate earlier. Data used to create the plot (including drug abbreviations) are in Supplementary Table S1.

**Figure 3:**
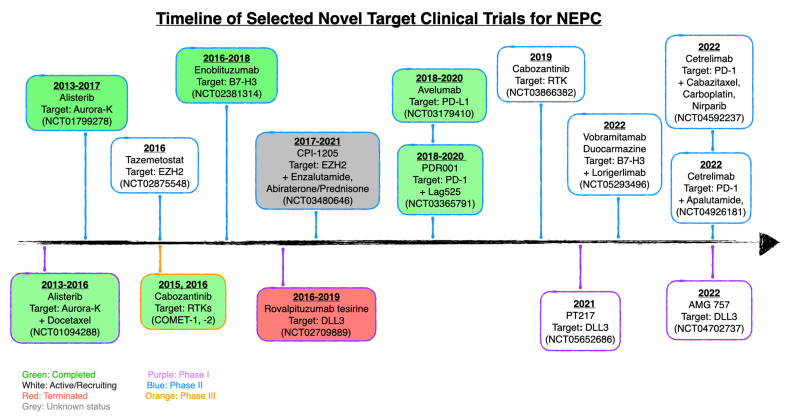
Timeline of selected novel target clinical trials for NEPC. Border colors indicate phase of the clinical trial (phase I, purple; phase II, blue; and phase III, orange). Fill colors indicate the status of the study (white, active/recruiting; green, completed; red, terminated; grey, unknown status).

The circos plot (Figure 2) visualizes drugs used in the reviewed combination trials for NEPC (Table 1). Additionally, Figure 3 provides a timeline of the NEPC trials discussed in this review. Among the 23 trials identified (Table 2), the plot reveals that chemotherapy and ADT are commonly used in combination with other therapies. This highlights the principle that decreasing androgen levels remains important even in the use of other targeting therapies, which has been shown in multiple clinical trials for MCRPC [77,78,79,80]. 

### 2.2. Novel Targets for Neuroendocrine Prostate Cancer

In this section, the novel targets that are associated with NEPC are identified and presented with their ongoing or recently completed clinical trials. 

#### 2.2.1. Target: DLL3

Delta-like ligand 3 (DLL3) is a negative regulator of the Notch signaling pathway and has been observed to be upregulated in NE tumors, including NEPC [81]. Furthermore, low DLL3 expression in normal prostate tissue makes it a promising therapeutic target [81]. Rovalpituzumab tesirine (SC16LD6.5) is an antibody-drug conjugate that specifically targets cells expressing DLL3; it consists of a monoclonal antibody against DLL3 coupled to a pyrrolobenzodiazepine warhead [82]. Originally tested in treatment resistant small cell lung cancer [83], (NCT01901653, NCT02674568) it was expanded into a phase I basket trial for treatment resistant metastatic solid tumors, including NEPC (NCT02709889). 

In a specific clinical case within the larger trial, a patient undergoing treatment with SC16LD6.5 received a dosage of 0.3 mg/kg every 6 weeks (NCT02709889). Subsequent scans following the first treatment cycle demonstrated significant clinical improvement, with a marked reduction in target nodal metastases from 42 to 24 mm and notable complete and partial responses observed in nontarget lesions. Imaging after the second treatment cycle revealed sustained stability, maintaining the nodal metastases at 24 mm, with no new lesions detected [81]. In this trial, the clinical benefit rate was 76.2% for all 21 participants with NEPC (NCT02709889). Clinical benefit rate was defined as a disappearance of tumor lesions, a decrease in diameter of tumor lesions, or no sufficient increase in the number of lesions indicating disease progression. The PFS was an average of 4.5 months with an OS average of 5.7 months (NCT02709889). Current ongoing phase I trials determine the safety, efficacy, and tolerability of DLL3-targeting drugs PT217 (NCT05652686) and AMG757 (NCT04702737). 

#### 2.2.2. Target: EZH2

Recent findings have implicated epigenetic dysregulation of transcriptional networks in therapy-induced lineage reprogramming to NEPC [52,84,85,86,87]. Atypical DNA methylation patterns and changes in the expression of epigenetic modifiers such as EZH2 (enhancer of zeste-homolog 2), transcription factors, and RNA-modifying factors are characteristic features of NEPC tumors [88]. EZH2 is a histone methyltransferase that induces transcriptional repression via methylation, and inhibition of EZH2 activity is being investigated as a novel target in clinical trials for NEPC. It has been shown to form a complex with N-Myc, a well-known oncogene in NEPC, and bind the androgen response element to downregulate AR target genes thereby maintaining the NEPC phenotype [89]. There are two ongoing studies assessing EZH2 as a novel target in clinical trials for NEPC, one using the drug CPI-1205, the small molecule inhibitor, in combination with Enzalutamide or Abiraterone/Prednisone (NCT03480646) and a long-term safety trial using the selective EZH2 inhibitor Tazemetostat (NCT02875548). 

#### 2.2.3. Target: B7-H3

B7-H3 belongs to the B7 family of ligands, which regulate cytotoxic T cell function and helper T cell function. B7-H3 has been found to have both co-inhibitory and co-stimulatory functions; however, its role in modulating the tumor microenvironment remains controversial [90]. There is evidence that B7-H3 overexpression has been observed selectively in tumor cells compared to normal cells [90,91]. Further evidence shows that B7-H3 is overexpressed in CRPC and is likely to have lower expression in NEPC compared to adenocarcinoma [92,93]. On this basis, B7-H3 may be a potential target in CRPC. Several B7-H3 targeted therapies are in (pre)clinical phases for a range of cancer types, but none have been clinically approved yet [92]. Among these are phase I clinical trials targeting B7-H3 in patients with CRPC. One trial evaluated the efficacy of the combination therapy of Enoblituzumab (MGA271), an investigational monoclonal antibody targeting B7-H3 with Ipilimumab (the monoclonal antibody against CTLA-4; Yervoy) in PC expressing B7-H3 (NCT02381314). This study has been completed, but the results have not been published. Additionally, an active study that is currently recruiting assesses the use of the antibody-drug conjugate Vobramitamab duocarmazine (MGC018) in combination with Lorigerlimab on patients with advanced solid tumors, including CRPC (NCT05293496). MGC018 is a humanized B7-H3 monoclonal antibody tagged by a cleavable linker to the drug secoDUocarmycin hydroxyBenzamide Anzaindole (DUBA) and Lorigerlimab is a bispecific DARTS (Drug Affinity Response Target Stability) molecule. This therapeutic regimen uses a combination approach to target independent antigen binding sites, i.e., PD-1 and CTLA-4.

#### 2.2.4. Target: Aurora Kinase

The Aurora kinase family comprises serine/threonine protein kinases that play a pivotal role in regulating cell cycle and mitotic functions, thereby ensuring the fidelity of genetic information [94]. Inhibition of these kinases results in mitotic defects and eventual cell death, making them attractive targets in cancer therapy. Alisertib (MLN8237) is a selective Aurora kinase A (AURKA) inhibitor that has previously been tested in multiple cancers. In NEPC models, AURKA inhibition has been shown to abrogate N-Myc signaling and suppress tumor growth. This is potentially mediated by the disruption of N-Myc stabilization by AURKA [95]. 

These findings set the stage for a phase I clinical trial, NCT01094288. This trial assessed the safety and tolerability of Alisertib in combination with the chemotherapeutic Docetaxel for patients with advanced CRPC, including NEPC. The investigators treated a small number of patients with different doses and combination of Alisertib and Docetaxel, ranging from 10 to 40 mg of Alisertib and 60 to 75 mg/m^2^ of Docetaxel (NCT01094288). While many patients did not complete the trial, 6 out of 14 patients responded to the treatment. The disappearance of target and non-target lesions and the normalization of tumor marker levels (serum PSA) were reported in patients showing complete response. Those with partial response fell into a category of patients showing a ≥30% decrease in sum of longest diameter of target lesions in reference to baseline (NCT01094288). PFS ranged from 79 to 830 days, with an average of 263 days (approximately 8.3 months) for all dose combinations (NCT01094288). OS data were not released for the study. 

A phase II clinical trial, NCT01799278, evaluated the efficacy of Alisertib in patients with NEPC. Results from the trial revealed a median PSA of 1.13 ng/mL (range: 0.01–514.2), with 68% of patients exhibiting visceral metastases. The six-month radiographic progression-free survival (rPFS) was 13.4%, and the median OS was 9.5 months (range: 7.3–13) (NCT01799278). Despite not meeting the primary endpoint, exceptional responders with complete liver metastasis resolution and prolonged stable disease were observed, notably in tumors indicative of N-Myc and AURKA overactivity. From both trials, Alisertib is a promising candidate for late stage NEPC, providing clinically relevant stabilization of disease in a subset of patients.

#### 2.2.5. Target: PD-1 and PD-L1

In the context of NEPC clinical trials, there has been growing attention towards the efficacy of immune checkpoint blockers (ICBs), particularly those that interact with the programmed cell death protein-1 (PD-1) and its ligand, PD-L1. The receptor protein, PD-1, resides on the surface of T cells and acts as a checkpoint and regulator for T cell activity [96]. When PD-1 is activated by PD-L1, a signaling pathway is induced that inhibits T cell activation. Cancer cells that produce PD-L1 are protected from T cell attack, thus reducing the interaction with T cells, and preventing the autoimmune reaction [96]. The issue arises when cancer cells, including NEPC, secrete excessive amounts of PD-L1 to evade immune attack from T cells, thereby creating a conducive environment for the cancer cells to proliferate [97].

To increase immune activity against cancer cells, such as NEPC, trials are designed using drugs that target either PD-1 or PD-L1. Cetrelimab is an anti-PD-1 antibody that has ongoing trials for NEPC and other cancers. There are promising results regarding its ability to bind to PD-1 in vitro and in vivo, though current phase II trials are still being analyzed [98]. For this inhibitor, there are two ongoing clinical trials that include patients with NEPC. A phase II trial investigated the outcome of a combined therapy of Cetrelimab and the ARSI Apalutamide (NCT04926181). According to the study, there is an increased expression of AR in certain NEPC biopsies; this subset of NEPC cells with AR is considered AR^high^/NE^high^ PC cells [99]. Based on this information, they concluded that combining an AR blockade strategy using Apalutamide and the immune checkpoint blocker, Cetrelimab, could yield better results than Cetrelimab alone (NCT04926181). In a similar context, an ongoing phase II trial of Cetrelimab and Niraparib, a PARPi, is being assessed. This evaluation follows a preceding combination of Cabazitaxel, Cetrelimab, and Carboplatin, aiming to utilize the patient’s immune system to target aggressive variant PC cells (NCT04592237). 

Similar to Cetrelimab, the antibody PDR001 was tested in a phase II trial for NE tumors (NCT03365791). PDR001 binds to PD-1 on the surface of T cells to nullify the effects of PD-L1 produced by NEPC cells [100]. In this trial, PDR001 was used in combination with LAG525, an inhibitor targeting the T cell activator LAG3 (lymphocyte-activation gene 3), to assess its efficacy as a combination therapy (NCT03365791). From this trial, they noted a median PFS of 2.8 months (range: 2.6–3.1) from 75 participants with NEPC and an ORR of 9.3% for this population (NCT03365791). The preliminary data show modest results in terms of efficacy and justify further research into maximizing its effects against NEPC. 

Beyond PDR001 and Cetrelimab, the PD-L1 inhibitor, Avelumab, has also been tested clinically. Avelumab binds to PD-L1 that is produced by NEPC cells, dissembling its defensive mechanism against T cells [101]. A completed phase II trial used single agent Avelumab to determine its safety for patients with NEPC (NCT03179410). The data presented a median rPFS of 1.8 months (range: 1.6–2.0) and median OS of 7.4 months (range: 2.8–12.5) within 15 participants (NCT03179410). Like PDR001, Avelumab displayed modest results regarding safety and its ability to reactivate immune responses. Further investigations are warranted to elucidate its precise mechanisms of action, efficacy in combination therapies, and its impact on long-term survival for NEPC patients.

#### 2.2.6. Target: Receptor Tyrosine Kinases

Targeting receptor tyrosine kinases (RTKs) is a promising and emerging approach in PC therapy. The inhibitor Cabozantinib suppresses metastasis, angiogenesis, and oncogenesis by inhibiting RTKs (including VEGFR, AXL, RET, and MET) and is approved by the FDA to treat metastatic renal cell carcinoma and medullary thyroid cancer [24,102,103]. 

There are several clinical trials that have tested or are testing the use of Cabozantinib for PC, and there are a few specific to NEPC. A phase III trial, COMET-1, revealed that there were no significant differences in OS between Cabozantinib (n = 686) and treatment with the anti-inflammatory agent Prednisone (n = 346). This regimen was administered to patients post-treatment with Docetaxel and Abiraterone and/or Enzalutamide [103,104]. A companion study, COMET-2, compared the combination treatment of Cabozantinib with Prednisone and the chemotherapeutic Mitoxantrone. The trial of 119 participants underwent an early termination due to the initial OS results from COMET-1, but further statistical analysis found a prolonged OS over the treatment period (9 vs. 7.9 months) and significantly improved bone scan response of 31% vs. 5.2% [105]. In addition, a phase II ongoing trial, NCT03866382 is evaluating therapeutic combination of Cabozantinib and two immunotherapy drugs, Nivolumab and Ipilimumab. The National Cancer Institute is currently recruiting a large variety of patients with rare genitourinary tumors and metastatic diseases for the trial, including NEPC (NCT03866382). 

## 3. Discussion

### 3.1. Combination Trials

For this review, the ClinicalTrials.gov database and The International Clinical Trials Registry Platform were queried; the search terms included “metastatic NEPC”, “CRPC”, and “NEPC”. A total of 39 clinical trials that targeted patients with advanced metastatic PC were identified; among these, 21 trials tested drug combinations while 17 tested monotherapies. One trial (NCT05582031) was withdrawn. In the drug combination trials, 20 trials included patients diagnosed as NEPC (Table 1). The drugs, broadly classified by their functions, and the tested combinations are visualized here using a circos plot (Figure 2). It is not surprising to note that more than half the trials (11 of 21 trials) feature either ADT or chemotherapy drugs. The most tested drugs are the chemotherapeutic docetaxel (5 trials) and the ADT drug, abiraterone (4 trials). Abiraterone, in combination with prednisone, has been approved for the treatment of MCRPC and shows promising survival statistics in patients receiving this treatment [79,80].

### 3.2. Novel Target Trials

Of the clinical trials reviewed, the PD-1 and PD-L1 inhibitors had the least favorable outcomes. While there are many trials ongoing for ICBs, it is within reason to question if they are effective to treat NEPC. Results from the Avelumab (anti PD-L1 monoclonal antibody) trial for NEPC patients showed a PFS of only 1.8 months and an OS of 7.4 months (NCT03179410). Likewise, PDR001 (anti PD-1 monoclonal antibody, Spartalizumab) provided a modest 2.8-month long PFS and a small ORR of 9.3% (NCT03365791). Though unfortunate, it is not entirely surprising that these ICBs failed to produce meaningful results. There is mounting evidence that PC is an immune cold cancer, meaning it has an immunosuppressive microenvironment [106]. Currently, there are few methods to effectively reintroduce the immune response in PC models, prompting the question of whether it is more advantageous to devote time and resources towards improving PD-1 and PD-L1 inhibitors or provide resources to therapies with proven potential in treating late-stage PC.

Conceivably the most anticipated trials for CRPC were the phase III companion trials COMET-1 and -2 with Cabozantinib. However, the data were disappointing in that there was no significant difference in OS between Cabozantinib and Prednisone in COMET-1. COMET-2 revealed a marginal improvement, with a modest increase in OS by 1.1 months and a significantly improved bone scan response [103]. Despite that, there is supporting preclinical data that suggests Cabozantinib could potentially inhibit tumor growth in NEPC harboring MET alterations or increased expression levels [107,108]. Furthermore, since Cabozantinib suppresses tumor growth via inhibition of angiogenesis, this could be a viable option for combination trials [102]. Together, these studies suggest that careful recruitment of NEPC patients based on biomarkers or molecular features may be necessary for future clinical trials with Cabozantinib [105].

The DLL3-targeted experimental-antibody drug conjugate Rovalpituzumab tesirine also exhibited a significant clinical response with a PFS of 4.5 months and an OS of 5.7 months in patients with NEPC (NCT02709889). Although the results are modest, this encourages the field to continue investigating doses and combinations trials that would maximize PFS using DLL3-targeted drugs. However, this novel target is still in the phase I timeline and may yield better results with additional ongoing trials and larger subgroups. 

The most promising results among the trials analyzed in this review were observed with the selective AURKA inhibitor Alisertib, which demonstrated the longest PFS of 8.3 months (NCT01094288) and an OS of 9.5 months (NCT01799278). Remarkably, one patient exhibited a survival period exceeding two years from the initiation of Alisertib treatment. However, there remains a critical need for more comprehensive data to discern the factors contributing to the varying treatment response among patients treated with Alisterib. Exploring potential biomarkers or predictors is crucial to determine whether Alisertib will prove beneficial for individuals with NEPC. Further phase II trials of Alisertib may be warranted before progression to phase III.

Sadly, there is a dearth of phase IV clinical trials targeting late-stage NEPC. While promising drugs and studies have potential to reach this stage of testing, it is necessary that further investigations into NEPC treatment are conducted. This form of cancer presents unique challenges that, once understood, can lead to innovative measures in the treatment landscape of NEPC. Though not specific to NEPC, there is a phase IV trial currently being performed to identify an effective modality to treat MCRPC that was unresponsive to Docetaxel (NCT02485691). The trial monitors the outcome of continued chemotherapy or ADT after unsuccessful treatment with Docetaxel. Phase IV trials such as this are promising avenues of research that could be applied to NEPC. 

### 3.3. Necessity of Biomarkers for Prevention and Understanding PC

The current understanding of PC biomarkers requires improvement, both in terms of identifying key elements within an assay and interpreting the clinical implications. There are continuous advancements in our knowledge of PC biomarkers that provide a positive direction for future prognosis. Lately, the use of molecular genetics has greatly aided better identification and detection of PC biomarkers for individual patients, providing a better view for personalized treatment plans [109]. For instance, looking at the PSA levels of a patient, or a secondary biomarker that acts as an accurate readout for drug activity, could confer the optimal dosage and subsequent outcome. Accomplishing this would lead to better use of current treatment modalities against NEPC. In addition, the use of AR as a biomarker for CSPC has shown promise for tracking a patient’s response to treatment modalities, such as ADT [110]. There is hope that this marker could be useful in predicting NEPC outcome as well. In analyzing PC genomics, it has been found that a sizable portion of the patients have mutations in homologous recombination repair (HRR), particularly those with MCRPC. Individuals harboring this mutation are prime candidates for PARP inhibitor treatment [111]. Therefore, assessing this mutation in NEPC patients could offer a promising pathway to discover better therapeutic options. 

From these improvements unique challenges in incorporating new data into current and future PC therapies [112] inevitably arise. While recent advancements in understanding and utilizing PC biomarkers offer promising avenues for personalized treatment plans, the incorporation of these new data into current and future therapies presents unique challenges. The heterogeneity of PC, particularly influenced by lineage reprogramming, underscores the complexity of formulating effective personalized treatments [113]. Due to this, one molecular alteration is not enough to elucidate treatment response; there must be multiple reliable genetic biomarkers to formulate treatment, thus increasing the difficulty in designing personalized therapies. 

### 3.4. Landscape of NEPC: Knowledge and Therapy

It is lamentable that the primary treatments approved for NEPC are predominantly sourced from research of other cancers. There are currently no notable therapies that precisely target either de novo or treatment emergent NEPC that have successfully completed the phases of clinical trials or been FDA approved. The present therapies used to combat NEPC were originally found to aid in treating small cell lung cancer. Their commonality in neuroendocrine phenotype often allows for the use of these therapies for NEPC [114]. 

In addition, there are few studies analyzing the prevention of NE transformation in PC. There are two known mechanisms of NEPC transformation, de novo NEPC and treatment emergent NEPC. Post ADT, a subset of patients relapses into androgen insensitive NEPC, likely occurring because of the therapy [61]. Further, plasticity, lineage reprogramming into NEPC, and clonal heterogeneity present challenges in treating MCRPC [52,61,115]. This emphasizes the need for further clinical research on (a) treating MCRPC and (b) preventing disease progression to incurable NEPC. 

The combination therapy of treating NE transformation with PARP inhibitors and CDK4/6 inhibitors has shown promising results in vitro and in vivo. This preclinical data suggest potential clinical trial opportunities [116]. Following this, it was discovered that inhibiting Exportin 1 through the downregulation of SOX2 is capable of suppressing NE differentiation in both prostate and lung cancers. Thus, exportin 1 is a candidate for drug-targeted inhibition to prevent the development of NEPC [117]. Though this is only for treatment emergent NEPC and does not include de novo NEPC, it gives hope for potential prevention therapies. 

Regarding the molecular mechanisms regulating NEPC differentiation, there is limited knowledge about the origin of de novo NEPC and treatment (ADT) induced NEPC. Mapping the pathways that lead to the NEPC phenotype is critical for identifying ideal targets for treatment, prognosis, and prevention. It is notable that a recent study has identified a potential culprit for NEPC and CRPC development [118]. A novel interaction between long-noncoding RNA (lncRNA) and microRNA (miRNA) appears to have a crucial role in promoting NEPC and CRPC differentiation. They have the ability to suppress translation, therefore modifying gene expression and promoting cellular transformation [118]. Though currently undetermined, this may be important for the development of de novo NEPC and could signify potential therapeutic targets for preventing NEPC.

### 3.5. Limitations and Concerns

Regrettably, among the trials surveyed for this review, a number are not explicitly tailored for this subset of aggressive PC, failing to segregate NEPC-specific data from the broader pool of information collected from other cancer patients. Due to this, it is difficult to discern whether the results of the trials are accurate for an NEPC-exclusive cohort. In addition, each completed trial contains a list of all adverse events experienced by the participants. However, many of the data do not confirm if these events are treatment related. Many trials do not further distinguish between patients in their list of adverse events, making the extent to which each participant is affected unclear. Thus, there is an obstacle in understanding what information is relevant to review without additional guidance from the trial investigators. 

Furthermore, a general lack of consensus in the United States on clinical trials methodology contributes to misguided conclusions, which is further exacerbated by the absence of quality scientific communication and collaboration between investigators. Often, this gap in the field can be attributed to barriers such as funding, dominant pharmaceutical manufacturers, and the lack of valuable partnerships within the science community. While this issue remains large in the United States, it also extends on the global level to a wide range of opinions on various clinical approaches in oncology [119]. Likewise, the process of selecting participants for a clinical trial lends itself to tailor-made cohorts that align with the treatment. This approach raises a major dilemma, as it is unlikely that tailor-made cohorts are reflective of the real-world individuals who will be prescribed the treatment. Similarly, many patients sign up for clinical trials as a last resort and have highly progressed disease. This has the potential to skew the trial’s results away from what the general population may exhibit and denies an understanding of how effective the treatment is at each stage of the disease. 

The motivation guiding the selection of the control arm is another variable that might introduce distortion into the data from these trials. Depending on the effectiveness of a control arm, the resulting data can look drastically different between two investigators observing similar treatments and populations. In general, this discrepancy between trials can contribute to varied statistical outcomes and cause bias in their results.

The present economic state of PC therapies adds to concerns regarding affordability and equity. Unfortunately, many drugs that have shown promise are also ones that are unavailable for clinical trials and/or are financially out of reach. The price difference between an available drug and the most effective drug can be outstanding in its ability to financially cripple a patient. While there are trials that are optimistic in their drug’s efficacy, formidable barriers between the patient and restoration of their health remain.

## 4. Conclusions

The clinical approach in treating PC is defined on a standard precedent formulated by clinical trials and the existing body of knowledge on the disease. Our findings demonstrate that both the currently approved treatments and clinical trials offered for NEPC are extremely limited. Addressing the unique challenges posed by NEPC, especially with treatment emergent NEPC resistance, require NEPC-specialized clinical trials, a search for effective biomarkers, research in the development of NEPC, and a more profound grasp on how its biochemical mechanisms interact with drugs. Furthermore, clinical research and design must be collaborative, communicative, and pursued with great care among the scientific community. In striving for these goals, the prognosis and outcomes for patients with aggressive NEPC can be improved. 

## Figures and Tables

**Table 1:** Combination clinical trials for NEPC.

Trial Identifier	Status	Phase	Drug 1	Drug 2	Drug 3	Drug 4	Drug 5
NCT02485691	Completed	4	Enzalutamide	Abiraterone	Cabazitaxel	Docetaxel	
NCT00973882	Completed, no results posted	2	Carboplatin	Etoposide			
NCT00014456	Completed, no results posted	1	Filgrastim	Docetaxel	Gemcitabine Hydrochloride		
NCT04848337	Recruiting	2	Pembrolizumab	Lanvatinib			
NCT03910660	Active, not recruiting	1/2	BXCL701	Pembrolizumab			
NCT04926181	Active, not recruiting	2	Apalutamide	Cetrelimab			
NCT03582475	Completed, no results posted	1	Pembrolizumab	Carboplatin	Cisplatin	Docetaxel	Etoposide
NCT02893917	Active, not recruiting	2	Cediranib	Olaparib			
NCT04592237	Recruiting	2	Cabazitaxel	Carboplatin	Cetrelimab	Niraparib	Cetrelimab (again)
NCT03902951	Active, not recruiting	2	Abiraterone	Apalutamide	Leuprorelin	Stereotactic Body Radiation Therapy	
NCT05582031	Withdrawn	2	Regorafenib	Tislelizumab			
NCT03649841	Terminated due to low accrual.	2	Antiandrogen therapy (unclear which drug/method was used)	Abiraterone	Prednisone	Radiation Therapy	
NCT05000294	Recruiting	1/2	Atezolizumab	Tivozanib			
NCT03333616	Active, not recruiting	2	Nivolumab	Ipilimumab			
NCT03896503	Active, not recruiting	2	Topotecan	Berzosertib			
NCT03365791	Completed	2	Spartalizumab (PDR001)	Leramilimab			
NCT03866382	Recruiting	2	Nivolumab	Ipilimumab	Cabozantinib		
DRKS00004797	Completed, no results posted	Unknown	Docetaxel	Cabazitaxel			
NCT03480646	Unknown status	1/2	CPI-1205	Enzalutamide	Abiraterone	Prednisone	
NCT02381314	Completed	1	Enoblituzumab (MGA271)	Ipilimumab			
NCT05293496	Recruiting	1/2	Vobramitamab Duocarmazine (MGC018)	Lorigerlimab			
NCT01848067	Completed	1	Alisertib (MLN8237)	Docetaxel			

**Table 2:** Selected novel target clinical trials for NEPC.

Trial Identifier	Status	Phase	Drug/Treatment	Target	Additional Drugs	NEPC Exclusive Cohorts
NCT02709889	Terminated	1	Rovalpituzumab tesirine (SC16LD6.5)	DLL3		Yes
NCT05652686	Recruiting	1	PT217	DLL3		Yes
NCT04702737	Active, not recruiting	1	AMG 757	DLL3		Yes
NCT03480646	Unknown status	1/2	CPI-1205	EZH2	Enzalutamide, Abiraterone/ Prednisone	Yes
NCT02875548	Active, not recruiting	1/2	Tazemetostat	EZH2		No
NCT02381314	Completed	1	Enoblituzumab (MGA271)	B7-H3	Ipilimumab	No
NCT05293496	Recruiting	1/2	Vobramitamab Duocarmazine (MGC018)	B7-H3	Lorigerlimab	No
NCT01094288	Completed	1	Alisertib (MLN8237)	Aurora-A	Docetaxel	No
NCT01799278	Completed	2	Alisertib (MLN8237)	Aurora-A		Yes
NCT03179410	Completed	2	Avelumab	PD-L1		Yes
NCT03365791	Completed	2	PDR001	PD-1	Lag525	No
NCT04926181	Active, not recruiting	2	Cetrelimab (JNJ-63723283)	PD-1	Apalutamide	Yes
NCT04592237	Recruiting	2	Cetrelimab (JNJ-63723283)	PD-1	Cabazitaxel, carboplatin, followed by nirparib	Yes
NCT03866382	Recruiting	2	Cabozantinib	Receptor Tyrosine Kinases		Yes
COMET-1, -2	Completed	3	Cabozantinib	Receptor Tyrosine Kinases		No

## Data Availability

Data sharing is not applicable.

## Supplementary Materials

The following supporting information can be downloaded at: https://www.mdpi.com/article/10.3390/ph17030351/s1, Table S1: Drug-target data used in Figure 2.

## Abbreviations

Prostate Cancer (PC), Prostate Specific Antigen (PSA), Digital Rectal Exam (DRE), Neuroendocrine Prostate Cancer (NEPC), Androgen Deprivation Therapy (ADT), Small Cell Neuroendocrine Carcinoma (SCNC), Large Cell Neuroendocrine Carcinoma (LCNEC), Computed Tomography (CT), Positron Emission Tomography (PET), Prostate-Specific Membrane Antigen (PSMA), Biochemical Recurrence (BCR), Castration Sensitive Prostate Cancer (CSPC), Castration Resistant Prostate Cancer (CRPC), Luteinizing Hormone-Releasing Hormone (LHRH), Androgen Receptor Signaling Inhibitors (ARSIs), Poly ADP Ribose Polymerase (PARP), Newly Diagnosed Metastatic Prostate Cancer (NDMPC), Overall Response Rate (ORR), Progression Free Survival (PFS), Overall Survival (OS).
